# Identification of aneuploidy-related gene signature to predict survival in head and neck squamous cell carcinomas

**DOI:** 10.18632/aging.205221

**Published:** 2023-11-20

**Authors:** Yu Liu, Yonghua Yuan, Tao Chen, Hongyi Xiao, Xiangyu Zhang, Fujun Zhang

**Affiliations:** 1 Department of Pharmacy, The First Affiliated Hospital of Chongqing Medical University, Chongqing 400016, China; 2 Research Center for Pharmacodynamic Evaluation Engineering Technology of Chongqing, College of Pharmacy, Chongqing Medical University, Chongqing 400016, China; 3 Department of Oral and Maxillofacial Surgery, the First Affiliated Hospital of Chongqing Medical University, Chongqing 400016, China

**Keywords:** head and neck squamous cell carcinomas, aneuploidy, ARS model, nomogram, therapy evaluation

## Abstract

Background: To parse the characteristics of aneuploidy related riskscore (ARS) model in head and neck squamous cell carcinomas (HNSC) and their predictive ability on patient prognosis.

Methods: Molecular subtyping of HNSC specimens was clustered by Copy Number Variation (CNV) data from The Cancer Genome Atlas (TCGA) dataset applying consistent clustering, followed by immune condition evaluation, differentially expressed genes (DEGs) analysis and DEGs function annotation. Weighted gene co-expression network analysis (WGCNA), protein-protein interaction, Univariate Cox regression analysis, least absolute shrinkage and selection operator (LASSO) and stepwise multivariate Cox regression analysis were implemented to construct an ARS model. A nomogram for clinic practice was designed by rms package. Immunotherapy evaluation and drug sensitivity prediction were also carried out.

Results: We stratified HNSC patients into three different molecular subgroups, with the best prognosis in C1 cluster among 3 clusters. C1 cluster displayed greatest immune infiltration status. The most DEGs between C1 and C2 groups, mainly enriched in cell cycle and immune function. We constructed a nine-gene ARS model (ICOS, IL21R, CCR7, SELL, CYTIP, ZAP70, CCR4, S1PR4 and CD79A) that effectively differentiates between high- and low-risk patients. Patients in low ARS group showed a higher sensitivity to immunotherapy. A nomogram built by integrating ARS and clinic-pathological characteristics helped predict clinic survival benefit. Drug sensitivity evaluation found that 4/9 inhibitor drugs (MK-8776, AZD5438, PD-0332991, PHA-665752) acted on the cell cycle.

Conclusions: We classified 3 molecular subtypes for HNSC patients and established an ARS prognostic model, which offered a prospective direction for prognosis in HNSC.

## INTRODUCTION

Head and neck cancer rank among the top six various cancers globally [1]. 90% of head and neck cancers are subdivided into HNSC and approximately 75% of these patients are related to alcohol consumption and smoking habit [2, 3]. Studies have shown that human papillomavirus is a risk indicator for HNSC [4]. More than half a million cases of people are diagnosed with HNSC annually [5]. Surgical excision, radiotherapy, administer medications or combination therapy have been widely adopted for HNSC treatment [6–8]. About 30% - 40% of HNSC patients diagnosed with early-stage could possess a 5-year survival of 70% - 90% followed by therapy, nevertheless, numerous HNSC patients are diagnosed at advanced stages. As a result, even individuals who get good health care have a survival rate of 30% - 40% [9]. Therefore, precision of early diagnostic detection is important for HNSC prognosis.

When chromosomes are not separated correctly during meiosis or mitosis, chromosomal aneuploidy will occur, in other words, cells carry increased or decreased individual chromosomes [10]. Aneuploidy, is also defined as somatic cell copy number alteration. Despite the harmful influence of aneuploidy in the process of life development, aneuploidy is prevalent in other fields, particularly cancers. The majority of solid tumors are aneuploid; however, cancer cells can endure chromosomal imbalance, which contributes to tumor evolution [11]. Aneuploidy is often related to proliferation genes, somatic mutation, and TP53 mutation [12]. Yet aneuploidy shows an inverse relationship with expression level of immune signaling genes [13]. A pan-cancer aneuploidy study revealed that squamous cancers possess a specific pattern of aneuploidy. Chromosome arm 3p loss and chromosome arm 3q gain, which exist in human papillomavirus positive or negative HNSC squamous tumors, are predominant characteristics of the squamous cancer cluster [13]. A recently published paper by William Jr. et al. further discovered that immune escape in HNSC precursor lesions transition is motivated by chromosome 9p loss [14]. Unfortunately, there is limited research on genes related to aneuploid in HNSC.

Here, we integrated aneuploidy-related genes in The Cancer Genome Atlas (TCGA)-HNSC cohort. A series of bioinformatics analysis were applied to construct a robust aneuploidy related riskscore (ARS) model to help predict HNSC clinical prognosis. To make full use of the complementary diagnostic value of clinicopathological characteristics, the ARS model was coupled with independent clinicopathological characteristics to establish a nomogram, facilitating ameliorative assessment of HNSC prognosis. We aimed to offer a prospective direction for prognosis in HNSC.

## MATERIALS AND METHODS

### Data collection and preprocessing for HNSC

First, gene expression profiles and clinical information on HNSC were obtained from the TCGA database (https://portal.gdc.cancer.gov/), which includes samples from 500 HNSC patients. Subsequently, we obtained genomic aneuploidy scores about the samples in TCGA-HNSC based on previous studies [13]. The “GDCquery” function of the R package “TCGAbiolinks” was used to download copy number variation (CNV) data for TCGA-HNSC, including data on important regions of gene amplification and deletion. For validation, GSE41613 [15] was obtained from Gene Expression Omnibus (GEO) database (https://www.ncbi.nlm.nih.gov/geo/). An ARS model on the predictive ability of immunotherapy was calculated using IMvigor210 from website (http://research-pub.gene.com/IMvigor210CoreBiologies/), GSE135222 [16] and GSE91061 [17], which are immune-treated datasets. Finally, the data preprocessing for TCGA-HNSC and GSE41613 cohorts contained: excluding samples without survival time and status, clinical follow-up information; ensembls and probes were uniformly converted into Gene symbol. The probe was removed if one probe matched with multiple genes.

### Consistency clustering and differentially expressed genes analysis based on CNV data

Package “ConsensusClusterPlus” [18] was used to perform cluster analysis applying CNV data as input. The main parameter settings were displayed below: maxK=10, reps=500 pItem=0.8, pFeature=0.8, clusterAlg=‘pam’ and distance=‘pearson’. The optimum cluster number was chosen by the cumulative distribution function (CDF). Kaplan-Meier analysis was executed to compare the overall survival (OS) between clusters. Thereafter, “limma” package [19] was employed to analysis DEGs between molecular clusters. DEGs were filtered with threshold |log2FC| > log2 (1.2) and false discovery rate (FDR) < 0.05.

### Immune condition evaluation

The proportions of 64 immune and stroma cell categories in each specimen were inferred applying the xCell package [20]. The xCell algorithm was used to transform gene expression levels into enrichment scores. For a supplement, the CIBERSORT algorithm [21] referring to LM22 dataset [22], was specially used to calculate the ratios of 22 immune cells. Lastly, general stroma level (StromalScore), immunocyte infiltration (ImmuneScore), as well as combination (ESTIMATEScore) of samples were calculated by Estimation of Stromal and Immune cells in Malignant Tumors using Expression data (ESTIMATE) [23].

### Weighted gene co-expression network analysis (WGCNA)

With the above analysis result, we used WGCNA to identify core modules related to aneuploidy and immunity applying DEGs. Firstly, we extracted the expression profiles of DEGs from TCGA-HNSC containing 500 specimens. We then calculated the distance between each gene using Pearson method, and established a weighted co-expression network using R package WGCNA [24]. The general module construction process was as follows. (1) The “pickSoftThreshold” function was adopted to screen soft thresholding. When the scaleless topological fitting index reached 0.9, the appropriate soft thresholding was determined; (2) the expression matrix is converted into an adjacency matrix, which was then transformed into a topological matrix. Then, hierarchical clustering method was employed for gene clustering, with the minimum number of genes in each gene network module of 30; (3) the eigengenes of each module were calculated, and then cluster analysis was performed to merge close modules into a new module.

### Functional enrichment analysis and protein-protein interaction (PPI)

The function of DEGs among molecular clusters was analyzed via Gene Ontology (GO) and Kyoto Encyclopedia of Genes and Genomes (KEGG) analysis via WebGestaltR (V0.4.4) R package [25]. The ggplot2 R package [26] was performed to draw a bubble diagram. The Search Tool for the Retrieval of Interacting Genes database [27] was executed to build a PPI network, which was visualized by Cytoscape3.10.1.

### Construction and estimation of a prognostic model and validation

The hub genes unrelated to HNSC prognosis were preliminarily excluded by performing Univariate Cox regression analysis. Then prognostic genes for HNSC were carried out for LASSO and stepwise multivariate Cox regression analysis. The final screened genes were used to build the model via Equation (1).


ARS=∑Coefficient i× Exp i

The i here represents the selected gene. Exp i represents the expression level of prognostic related gene.

Given the “surv_cutpoint” function in survminer package [28], the best cutpoint was discovered and the HNSC patients were assigned into ARS high and low groups. Kaplan-Meier curves accompanied with Log-rank algorithm were performed for analysis of prognostic differences.

### Establishment and assessment of a nomogram

Originally, we structured a decision tree based on the Grade, T Stage, Age, Gender, N Stage, Clinic stage, and ARS of patients in the TCGA-HNSC cohort. Independent prognostic factors were screened by Univariate and Multivariate Cox regression analysis. Then, we utilized these independent prognostic factors to establish a nomogram via “rms” package [29]. Furthermore, we use the calibration curve to evaluate the predictive accuracy of the nomogram. Additionally, we also evaluated the reliability of the nomogram applying decision curve.

### HNSC cell line and drug sensitivity analyses

Drug sensitivity data of about 1000 cancer cell lines were downloaded from Genomics of Drug Sensitivity in Cancer (GDSC) (http://www.cancerrxgene.org). Taking the antitumor drug area under concentration-time curve (AUC) in the HNSC cell line as the drug response index, the correlation between AUC and ARS was calculated by Spearman correlation analysis. |Rs| > 0.3 and FDR < 0.05 were considered significantly correlated. Simultaneously, drug sensitivity differences between risk groups were compared. In addition, we also acquired the AUC values of 1037 cell lines from The Cancer Cell Line Encyclopedia (CCLE) website (https://portals.broadinstitute.org/ccle/) [30], screened the cell lines of HNSC, including 504 cell lines treated with 24 drugs, and performed correlation and difference analysis.

### Statistical analysis

Statistical analyses were performed using R software accompanied with Sangerbox website (http://sangerbox.com/) [31]. *P* < 0.05 was regarded as statistically significant. Kaplan–Meier survival curves for survival analysis were plotted applying log-rank algorithm. The correlation among genes, infiltrating immune cell and aneuploidy score was determined using Spearman method.

## RESULTS

### Molecular subgroup correlation analysis based on CNV data

**Figure 1:**
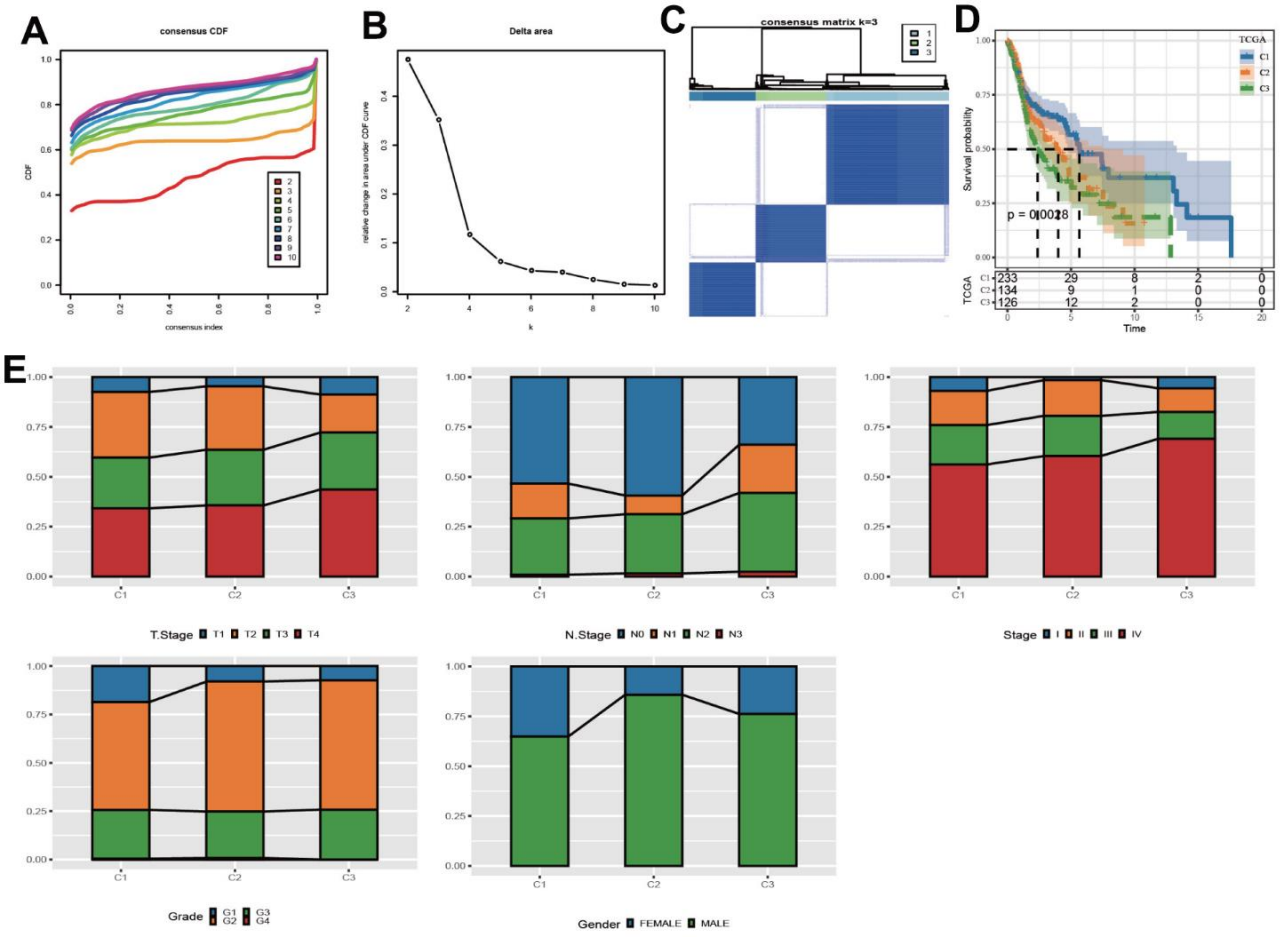
**Identification of molecular subpopulations based on CNV data.** (**A**) CDF curve of samples at different k values in the TCGA cohort. (**B**) CDF Delta area curves. (**C**) Heatmap of clustering result when k=3. (**D**) Kaplan-Meier analysis. (**E**) The distribution of clinical features in 3 clusters.

According to CDF Delta area curve, a relative stable clustering result was obtained when k=3 (Figures 1A, 1B). Therefore, the LUAD-HNSC cohort was assigned into three clear molecular subtypes, which we defined as C1, C2 and C3 (Figure 1C). Survival analysis revealed that C1 subgroup had the longest OS time, while C3 and C2 subgroups had relative shorter OS time (Figure 1D). In addition, to the differences between the three clusters in terms of clinically relevant characteristics, we found that in comparison with C1, both C2 and C3 showed advanced clinical stages and tumor grades, while not showing significant gender differences (Figure 1E).

### Characterization of the immune cell infiltrations in three subgroups

**Figure 2:**
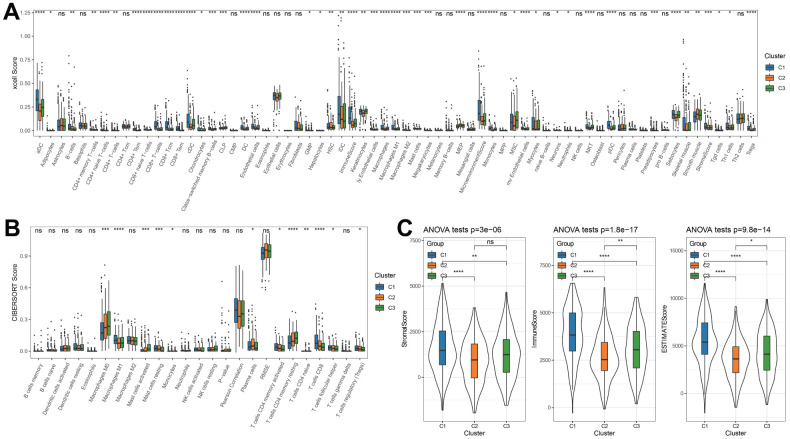
**Characterization of the immune infiltration in three clusters.** (**A**) XCELL analysis. (**B**) CIBERSORT analysis. (**C**) ESTIMATE analysis. *p < 0.05, **p < 0.01, ***p < 0.001, ****p < 0.0001, ns: no significance.

The XCELL analysis results discovered that B cells, aDC, cDC, iDC, CD8+Tem, CD8+ T-cells, sebocyte, Th1 cells, Tregs, CD8+ Tcm, StromalScore, MicroenvironmenScore had the highest score in C1 cluster than other two clusters (Figure 2A). Consistently, among 22 immunocytes, T cells CD8, Mast cells resting, tregs, T cells CD4 memory activated, and Macrophages M1 had the highest ratios in C1 cluster (Figure 2B). Through ESTIMATE analysis, a relatively higher StromalScore, ImmuneScore and ESTIMATEScore in C1 cluster also demonstrated higher inflammatory infiltration status and lower tumor abundance (Figure 2C).

### Differentially expressed genes and function analysis in three groups

**Figure 3:**
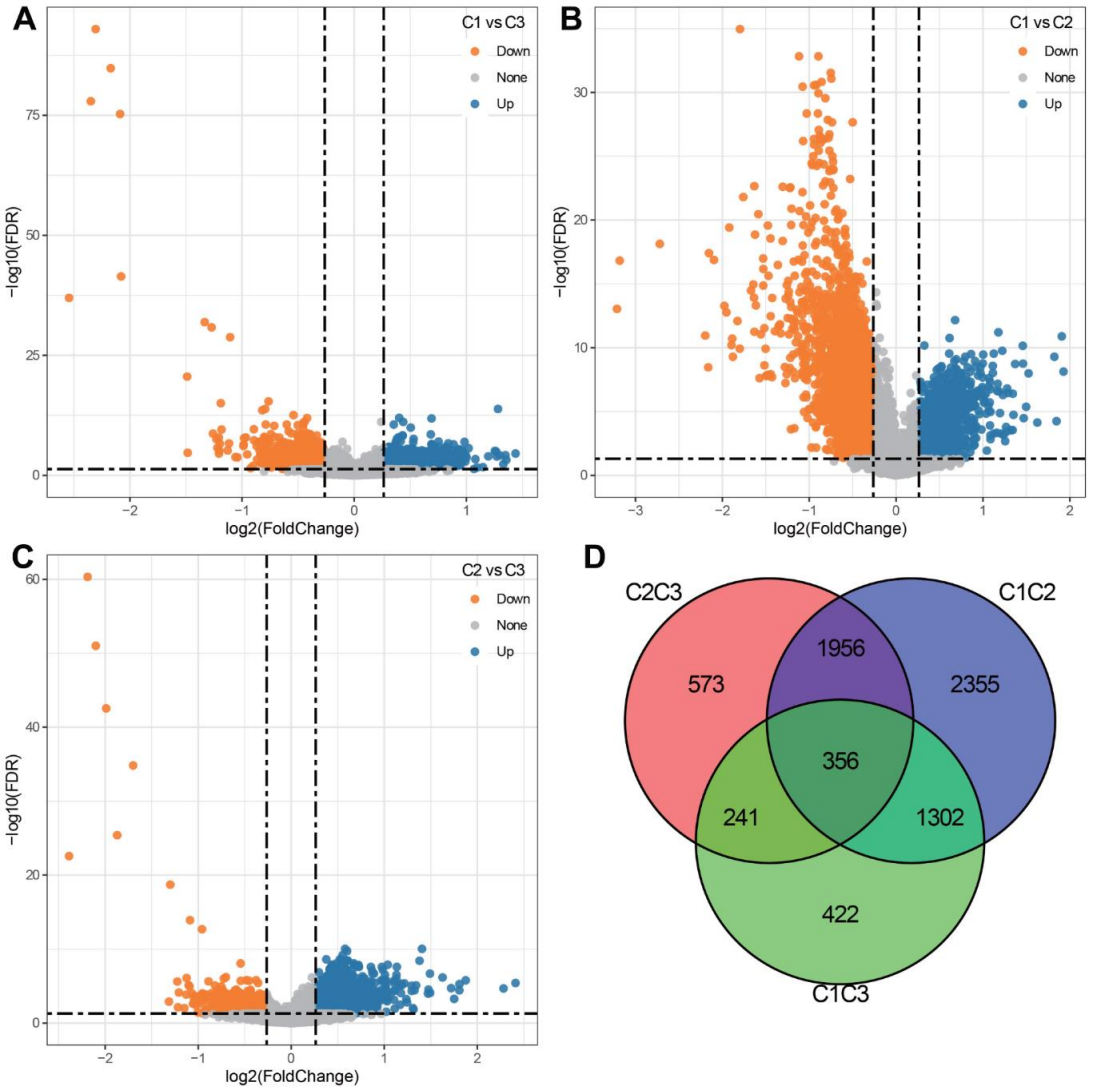
**Differentially expressed genes (DEGs) among 3 clusters.** (**A**) Volcano plot depicting DEGs between C1 and C3 groups (920 up-regulated and 1401 down-regulated). (**B**) Volcano plot depicting DEGs between C1 and C2 groups (1075 up-regulated and 4894 down-regulated). (**C**) Volcano plot depicting DEGs between C2 and C3 groups (2699 up-regulated and 427 down-regulated). (**D**) Venn diagram describing the intersection of DEGs among 3 clusters.

**Figure 4:**
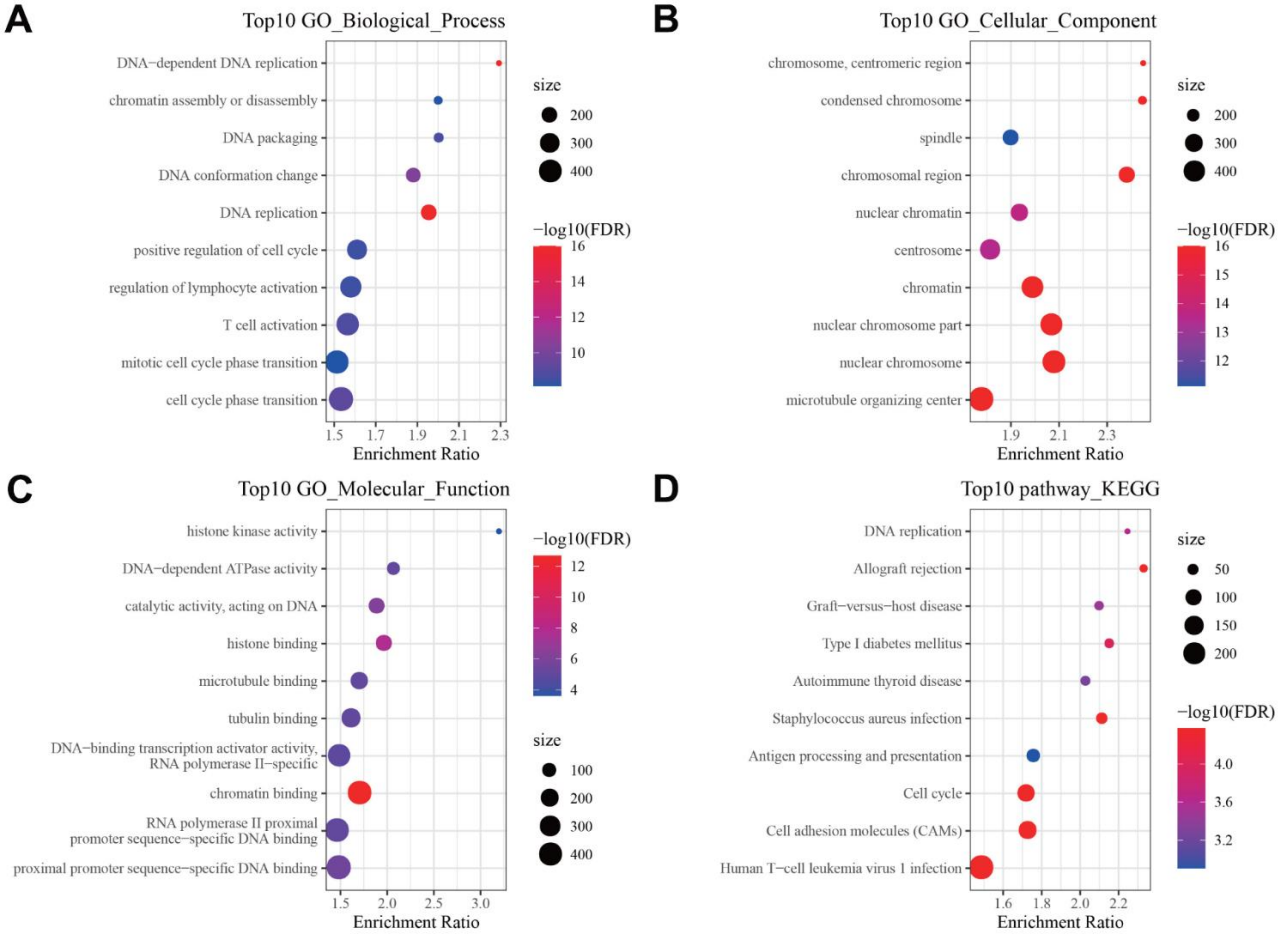
**Pathway enrichment analysis of DEGs.** Bubble diagram showing the top 10 enriched (**A**) Biological Processes, (**B**) Cellular Components, (**C**) Molecular Functions in GO annotation, and (**D**) KEGG pathways enriched by DEGs.

We compared the DEGs between three subtypes in pairs. As shown in Figure 3A–3C. 2321 DEGs were identified in C1 and C3 subgroups. 5969 DEGs were identified in the C1 and C2 subgroups. 3126 DEGs were identified in the C2 and C3 subgroups. By merging three sets of DEGs, a total of 356 shared DEGs were obtained (Figure 3D). Among 3 subgroups, the C1 and C2 subpopulations screened the most DEGs. We therefore conducted GO functional enrichment analysis and KEGG pathway analysis on these DEGs. GO enrichment analysis uncovered that DEGs were mainly enriched in Biological Process, containing DNA replication, immune response and cell cycle (Figure 4A); in the field of Cellular Component, DEGs were predominately centralized to the chromosome resign, spindle and microtubule organizing center (Figure 4B); with regard to Molecular Functions, DEGs were largely involved in DNA related activities, microtubule binding and chromatin binding (Figure 4C). KEGG pathway analysis displayed that DEGs also highly enriched in cell cycle and immune relevant signaling pathway or diseases (Figure 4D).

### WGCNA analysis based on DEGs

**Figure 5:**
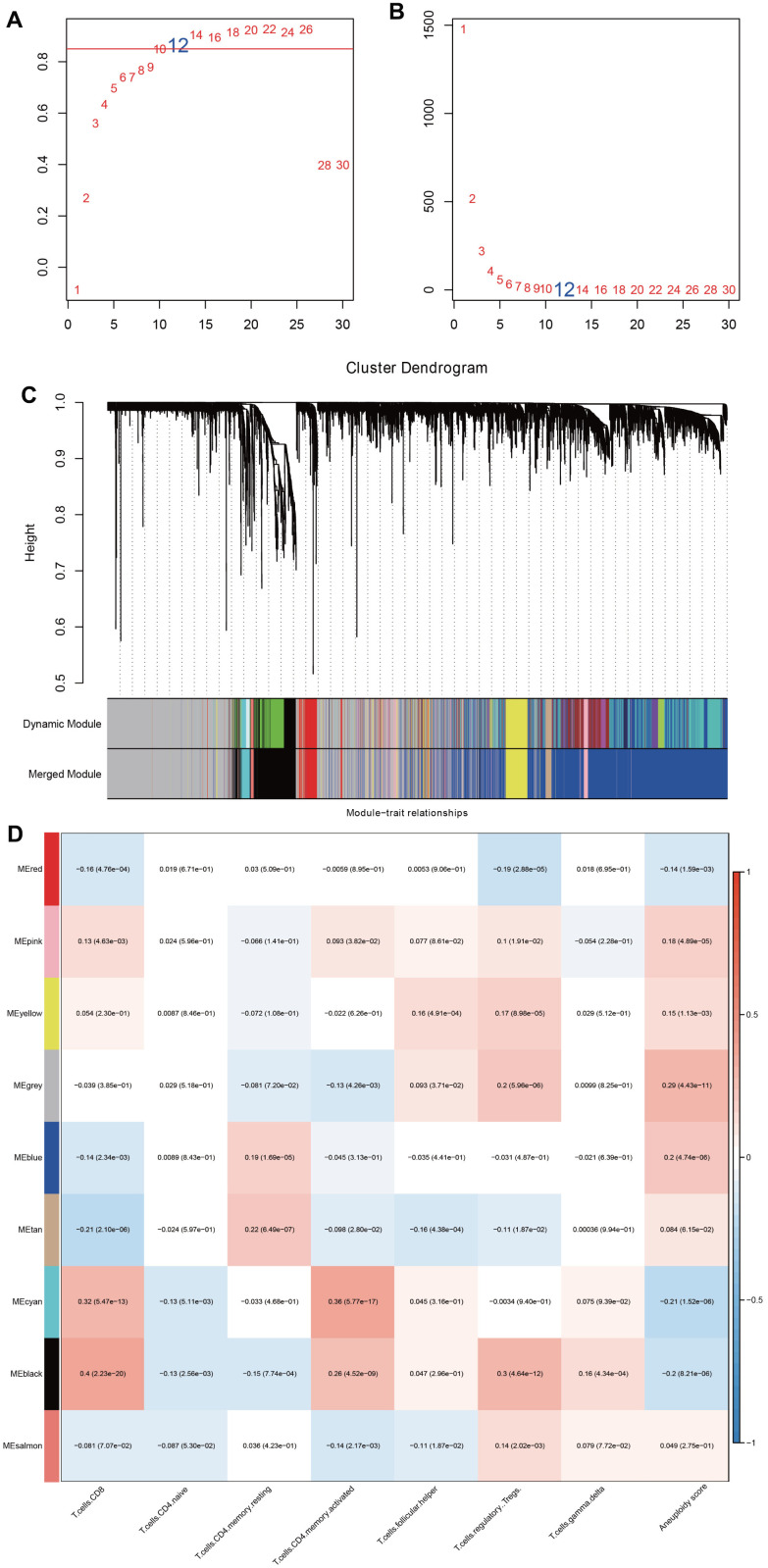
**Identification of key modules related to immune infiltration and aneuploidy by WGCNA.** (**A**, **B**) Analysis of network topology for various soft-thresholding powers. (**C**) The dendritic map of dynamic module and merged module. (**D**) Correlation analysis among merged module, immune infiltrating cells and aneuploidy score.

By calculation, when a soft threshold was chosen as 12, the degree of independence can reach 0.9, which showed good network connectivity (Figure 4A, 4B). After using the dynamic clipping method to determine the gene modules, we sequentially calculated the eigengenes of each module (Figure 5A, 5B). By setting deepSplit=2, mergeCutHeight=0.25, and minModuleSize=30, we obtained a total of 9 new modules (Figure 5C). It should be pointed out that the grey module is a gene set that cannot be aggregated to other modules. The correlation between each module and T cell infiltration and aneuploidy score was exhibited in Figure 5D. The black model displayed a positive correlation with immune infiltrating cells such as T cells CD8 and T cells memory activated, while showed an inverse correlation with aneuploidy score. Totally 515 genes were included in black models.

### Constructing a PPI interaction network for key module genes

**Figure 6:**
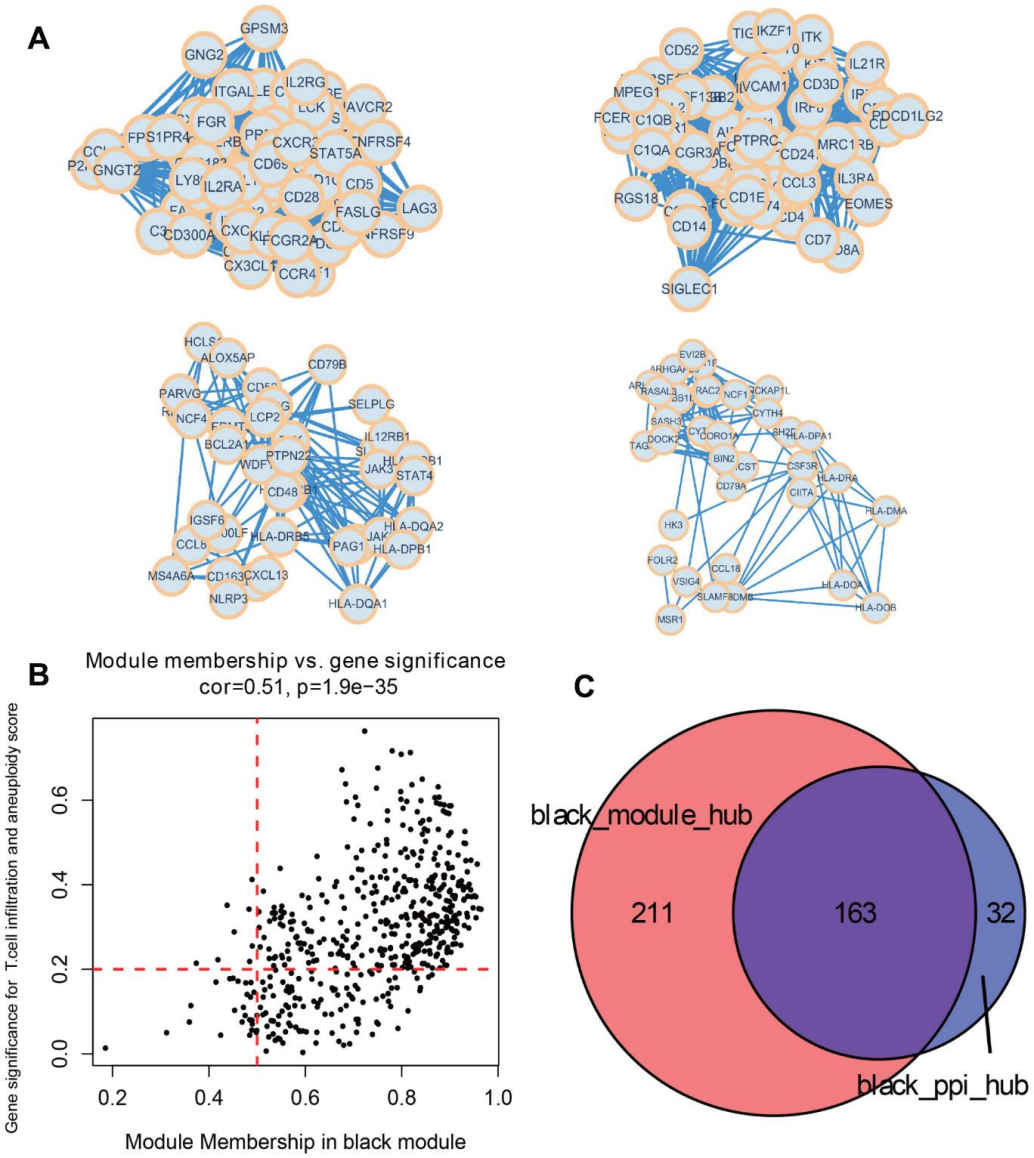
**Identify key module hub genes based on black module and PPI network.** (**A**) Four key protein groups of PPI interaction network constructed by genes in black module. (**B**) Scatter diagram for module membership vs. gene significance concerning immune infiltrating cells and aneuploidy score in the black module. (**C**) Venn diagram displaying the intersection of hub genes in the black module.

515 genes from the black module were used to construct a PPI interaction network. Based on MCODE algorithm, four tightly connected protein groups containing 195 genes were selected as the network hub genes (Figure 6A). At the same time, we used the black module, which were significantly correlated with both T cell infiltration and aneuploidy score, to select genes. In accordance with the screening requirements (Module Membership > 0.5 and Gene Signature > 0.2), a total of 374 significant genes were screened (Figure 6B). Taken together, a total of 163 identical hub genes were obtained by intersecting the significant genes with the hub genes from the PPI network (Figure 6C).

### Establishment and assessment of a prognostic signature

**Figure 7:**
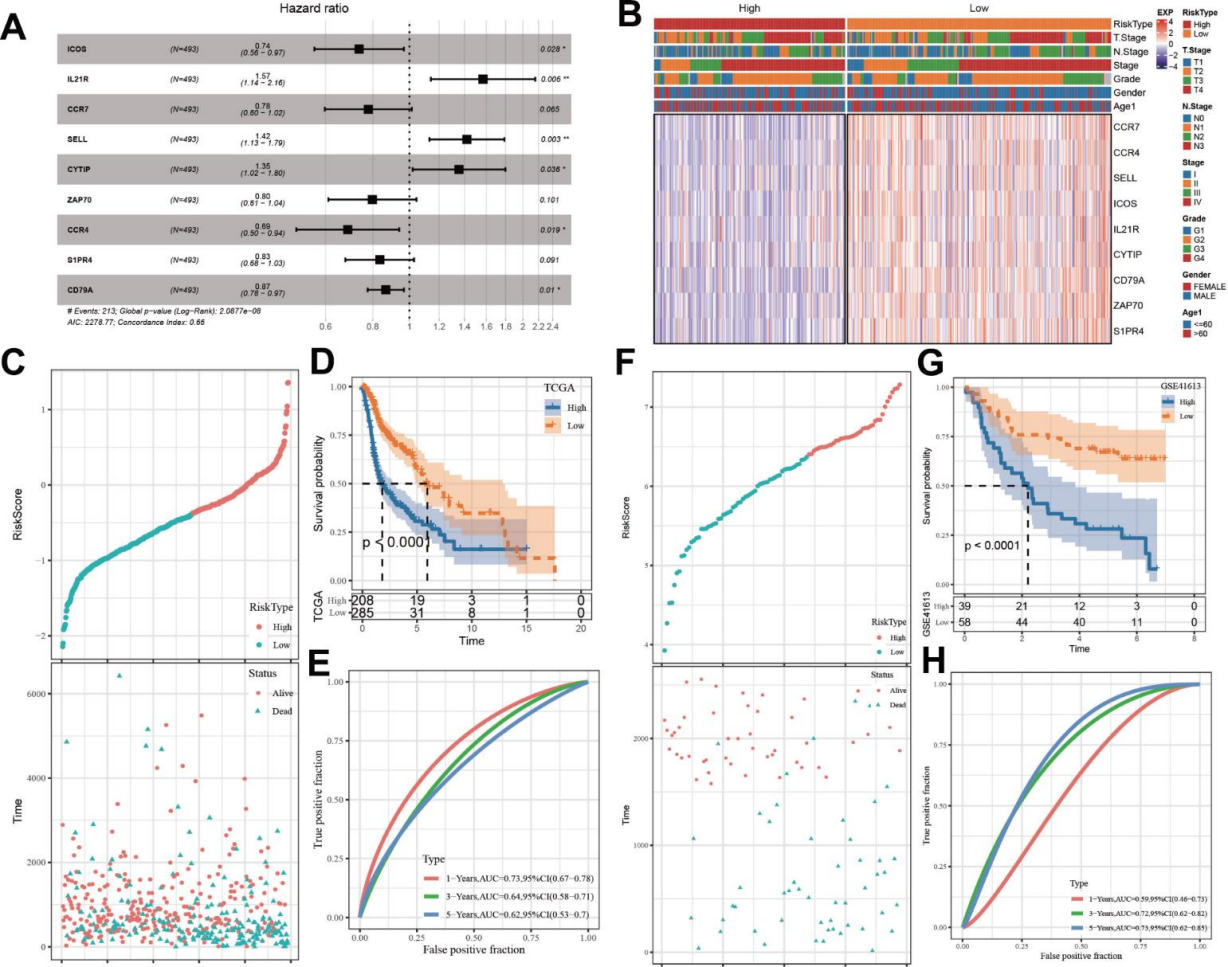
**Establishment and assessment of an ARS signature.** (**A**) Multivariate Cox analysis about 9 selected genes. (**B**) The expression differences of 9 selected genes in samples with different clinical characteristics. (**C**) The relationship of ARS with survival time and survival status in TCGA cohort. (**D**) Kaplan-Meier survival curve distribution of 9-gene signature in TCGA cohort. (**E**) Time-ROC analysis of 9-gene signature in TCGA cohort. (**F**) The relationship of ARS with survival time and survival status in GSE41613 queue. (**G**) Kaplan–Meier survival curve distribution of 9-gene signature in GSE41613 queue. (**H**) Time-ROC analysis of 9-gene signature in GSE41613 queue. *p < 0.05, **p < 0.01.

On the foundation of above 163 identical hub genes in the black module, we implemented Univariate Cox regression analysis and found 47 genes correlated with HNSC prognosis (p < 0.01, Supplementary Figure 1A), all of which were protective factors (Hazard Ratio < 1). 13 of the 47 genes were reserved by performing LASSO-Cox regression model with appropriate lambda (lambda = 0.0135) (Supplementary Figure 1B, 1C). Finally, 9 genes were determined to construct the model via stepwise multivariate regression analysis (Figure 7A). The expression differences of these 9 genes in various clinical features were described Figure 7B. Each patient’s ARS was calculated by Equation (2).


$$\begin{array}{lll} \text{ARS}=-0.307 & \times & \text{Exp}\;\text{ICOS}\\ & + & 0.45\times \text{Exp}\;\text{IL}21\text{R}\\ & - & 0.25\times \text{Exp}\;\text{CCR}7\\ & + & 0.351\times \text{Exp}\;\text{SELL}\\ & + & 0.303\times \text{Exp}\;\text{CYTIP}\\ & - & 0.225\times \text{Exp}\;\text{ZAP}70\\ & - & 0.376\times \text{Exp}\;\text{CCR}4\\ & - & 0.181\times \text{Exp}\;\text{S}1\text{PR}4\\ & - & 0.144\times \text{Exp}\;\text{CD}79\text{A} \end{array}$$

Given median ARS value, the patients were stratified into ARS high and low groups. Patients with high ARS displayed worse survival status (dead) and shorter OS time (Figures 7C, 7D), indicating that samples with high ARS have poorer prognosis. Time-dependent receiver operating characteristic analysis (ROC) analysis confirmed the predictive ability of the ARS model in HNSC disease, because all values of area under the curve were bigger than 0.6 (Figure 7E). The model got validated in GSE41613 dataset (Figure 7F–7H).

### ARS integrated with clinic indicators helped augment the survival evaluation of HNSC patients

**Figure 8:**
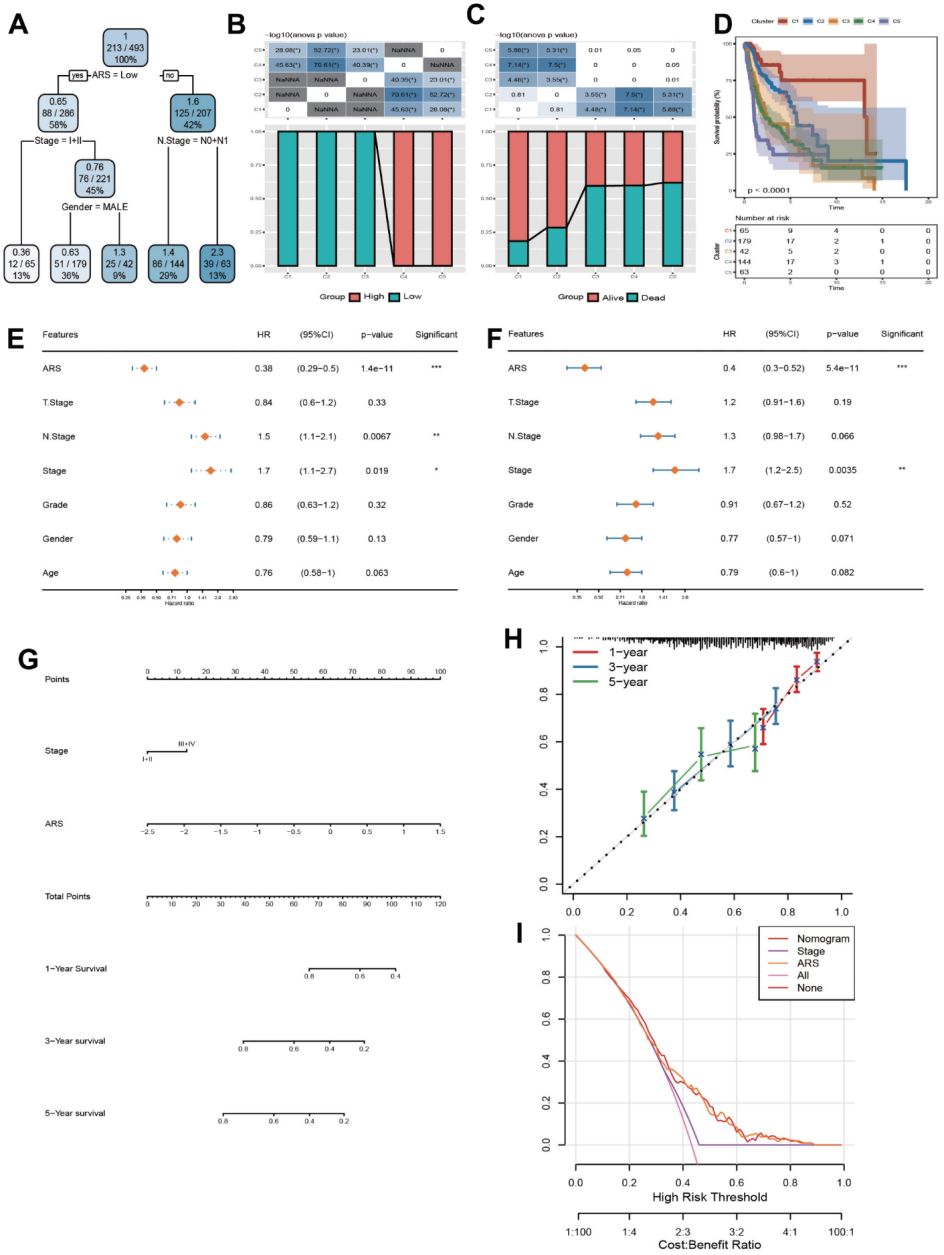
**Construction and validation of a nomogram.** (**A**) Construction of a decision tree. (**B**) Kaplan-Meier analysis for 5 subgroups. (**C**, **D**) Comparative analysis of high and low ARS population and survival status among 5 subgroups. (**E**, **F**) Univariate and Multivariate Cox regression analysis concerning ARS and clinic features. (**G**) Construction of a nomogram. (**H**) Calibration curve of the nomogram for 1, 3, and 5 years. (**I**) Decision curve of the nomogram.

The structure of decision tree was described in Figure 8A. 5 risk groups were determined. There were remarkable disparities in OS time and OS status among the five risk subgroups (Figure 8B, 8C). Among them, patients in groups C1, C2, and C3 all had low ARS, risk groups C4 and C5 only contained high ARS patients, yet (Figure 8D). Furthermore, ARS and Clinic stage were confirmed by Univariate and Multivariate Cox regression analysis as the independent prognostic indicators (Figure 8E–8F). Hence, a nomogram was formed with ARS and Clinic stage (Figure 8G). As displayed in Figure 8H, it can be observed that the predicted calibration curves for 1, 3, and 5 years were close to the standard ones, suggesting that the column chart had a strong predictive performance. In Figure 8I, the benefits of nomogram and ARS are significantly higher than the extreme curve, implying that nomogram and ARS both exhibited the strong survival prediction ability for clinical practice.

### Immunotherapy evaluation and drug sensitivity prediction

**Figure 9:**
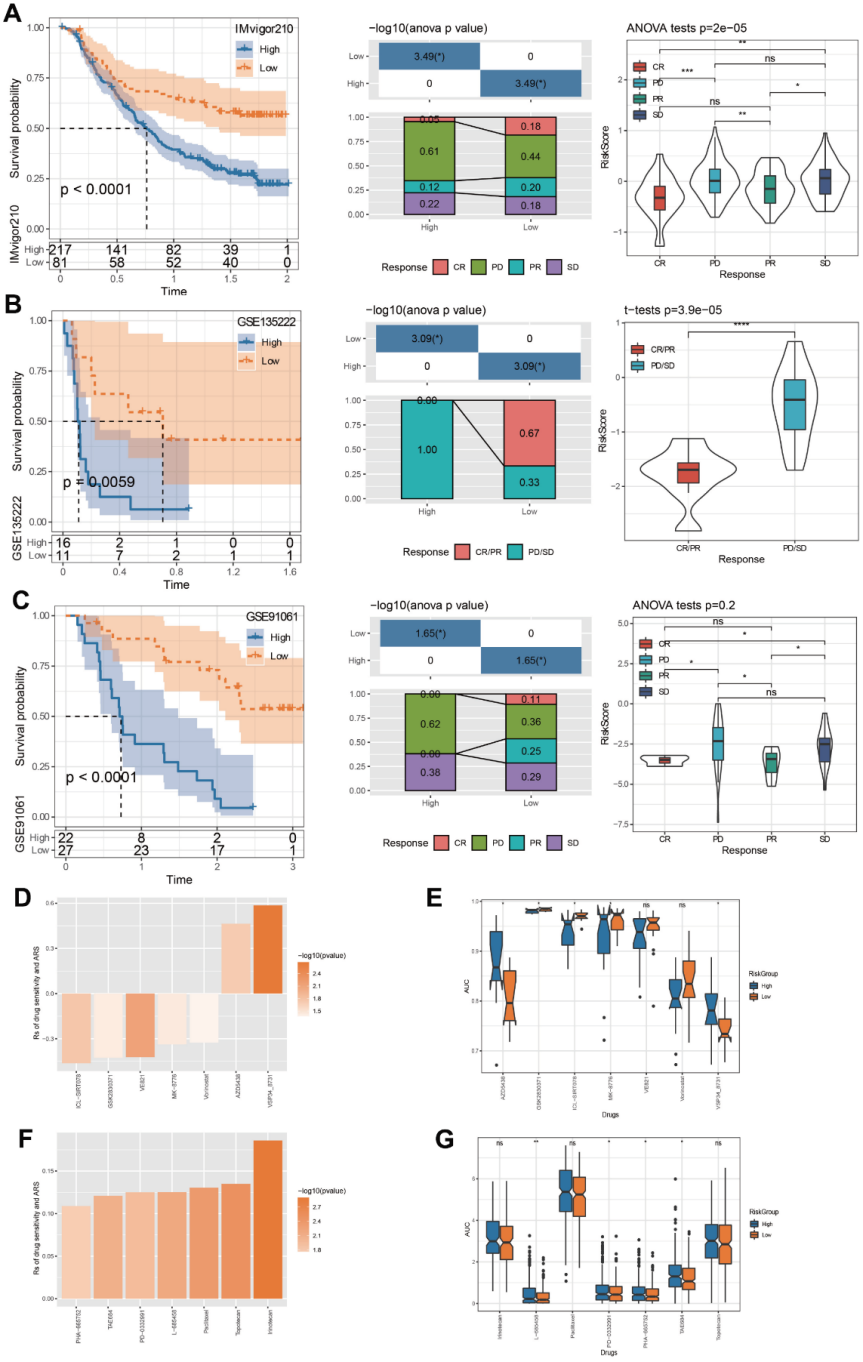
**Immunotherapy evaluation and drug sensitivity prediction.** (**A**) ARS survival curve and immunotherapy distribution in IMvigor210 dataset. (**B**) ARS survival curve and immunotherapy distribution in GSE135222 dataset. (**C**) ARS survival curve and immunotherapy distribution in GSE91061 dataset. (**D**) Correlation between TCGA-HNSC cohort ARS and drug AUC values in GDSC database. (**E**) The distribution of each drug’s AUC based on GDSC database between risk groups. (**F**) Correlation between TCGA-HNSC cohort ARS and drug AUC values in CCLE database. (**G**) The distribution of each drug’s AUC based on CCLE database between risk groups. *p < 0.05, **p < 0.01, ***p < 0.001, ****p < 0.0001, ns: no significance.

Tumor immunotherapy is considered an effective treatment for cancer [32]. In this study, the datasets IMvigor210, GSE135222, and GSE91061 were all immunotherapy treated data. Applying these data, we used our method to calculate ARS scores and predicted survival curves by plotting Kaplan-Meier curves with median cutoff. The newly defined low ARS group exhibited prolonged OS time, and the progressive disease (PD)/ stable disease (SD) was higher in the high ARS group (Figure 9A–9C).

Through the GDSC database, we screened that the drug AUC values of ICL-SIRT078, GSK2830371, VE821, MK-8776, Vorinostat, AZD5438 and VSP34_8731were correlated with ARS (Figure 9D). Chemotherapy has an important role in controlling tumor progression [33]. By comparing the drug sensitivity differences between risk groups, we found significant differences among the 5 drugs, among which high ARS patients are more sensitive to AZD5438 and VSP34_ 8731, and low ARS patients showed notable response to GSK2830371, ICL-SIRT078, and MK-8776 (Figure 9E). By means of the CCLE database, we observed that PHA-665752, TAE684, PD-0332991, L-685458, Paclitaxel, Topotecan, and Irinotecan, was significantly correlated with ARS (Figure 9F). In particularly, high ARS patients were more sensitive to L-685458, PD-0332991, PHA-665752, and TAE6844 (Figure 9G).

## DISCUSSION

Head and neck cancers constitutes approximately 90% of HNSC [2]. HNSC starts from the mucosal epithelium of the mouth, pharynx, and throat and has been thought as the most prevalent malignancies in the head and neck. Considering that the aneuploidy is a sign of tumor [34], investigating aneuploidy related biomarkers to evaluate the treatment effect of patients with HNSC is crucial. In this research, CNV data and mRNA expression data from HNSC specimens were derived from the TCGA database. Then, we performed consistency clustering analysis applying CNV data. For obtained 3 clusters, we elucidated the OS, immune inflammatory infiltration, function analysis and merged intersecting DEGs in 3 clusters. Subsequently, we conducted WGCNA, correlation analysis with regard to aneuploid and T cell infiltration based on the intersection DEGs to obtain the target module. Through further PPI analysis and Lasso analysis, we ultimately established an independent 9-gene ARS model. This signature helps offer a prospective direction for prognosis in HNSC.

Here, through a series of differential analysis, we established a robust ARS model including nine genes, namely ICOS, IL21R, CCR7, SELL, CYTIP, ZAP70, CCR4, S1PR4 and CD79A. Several researches have uncovered relationships between cancer tumorigenesis and pathogenesis and these genes. As a co-stimulatory receptor for T-cell enhancement, inducible Co-Stimulator (ICOS) has been regarded as a beneficial biomarker for immuno-oncology [35]. For example, Duhen et al. [36] discovered that PD-1 and ICOS co-expression helped recognize tumor responsive CD4+ T cells in HNSC immune infiltrating cells. Interleukin-21 receptor (IL-21R) was reported to take part in JAK/STAT signaling pathway and can activate anti-tumor immunity, depress inflammation and tumor occurrence [37]. An immune-related riskscore model constructed by Yao et al. demonstrated that high expressed IL21R, a protective indicator, displayed prolonged OS time in HNSC patients [38]. Consistently, in our research, IL21R was also overexpressed in low ARS group patients, who had favorable prognosis. The human CC chemokine receptors such as CCR4 and CCR7 involve in T cell trafficking [39]. Over expressed CCR4 appeared to be treated as a biomarker for immune checkpoint inhibitor therapeutic response in renal cancer patients [40]. Similar to our findings, a high-level of CCR4 was also correlated with good prognosis in HNSC suffers through joining in immune infiltration [41]. SELL is gene related to T cell stemness [42], which has been a research hotspot for enhancing cancer immunotherapy [43]. However, SELL was rarely mentioned in cancer studies. Cytohesin 1 Interacting Protein (CYTIP) was reported to exhibit sensitive response to anti-PD-1 therapy [44], implying its potentiality as responsive biomarkers for anti-PD-1 immunotherapy in non-small cell lung cancer. In another pan-cancer investigation, CYTIP was identified as an immunosenescence gene, that could also be regarded as a biomarker for immunotherapy in melanoma [45]. In a systematic immune genes analysis paper, ZAP70 was recognized as a prognostic immune gene, which was related to improved OS in HNSC [46]. A newly published study [47] and our findings both further augmented the role of ZAP70 in HNSC. In present research, low ARS group patients with good prognosis displayed highly expressed S1PR4, which seemed to be positively correlated with prognosis of HNSC. Yet, in other study, S1PR4 inhibition restrained tumor development accompanied with ameliorative chemotherapy [48]. The phenomenon maybe caused by tumor heterogeneity or differences in sample slices. In the cervical cancer relevant immune microenvironment analysis, both CCR7 and CD79A were selected as representative genes concerning survival outcomes [49]. Taken together, our results suggest that prognostic genes based on aneuploidy characteristics may be critical for the immune microenvironment and prognosis of HNSC. Abnormal number of chromosomes indicating genomic instability refer to aneuploidy, which often takes place in cell cycle [50]. In this study, we probed aneuploidy related genes in view of CNV data in TCGA-HNSC, and discovered an interesting phenomenon. The Biological Process in GO and KEGG enrichment analysis of DEGs between C1 and C2 molecular subtypes were mainly enriched in cell cycle function. In the same time, drug sensitivity evaluation based on ARS model found that 4/9 inhibitor drugs (MK-8776 [51], AZD5438 [52], PD-0332991 [53], PHA-665752 [54]) also acted on the cell cycle. These results supported the reliability of our research process.

Although we integrated ARS and few clinical features to construct a nomogram, which displayed satisfying predictive performance. The insufficient data in this study demand a large amount of clinical data for model calibration for future practical utilize.

## CONCLUSIONS

Our study established an aneuploidy-related gene signature for prognosis in HNSC patients. The ARS model displayed a favorable predictive capacity. ARS model can also assist patient’s immunotherapy and drug treatment, thus contributing to personalized precision treatment decisions for HNSC patients.

## Supplementary Material

Supplementary Figure 1
