# Supplementary material for: Identification of aneuploidy-related gene signature to predict survival in head and neck squamous cell carcinomas
Source: Aging (Albany NY). 2023 Nov 20;15(22):13100–17. doi: 10.18632/aging.205221 (PMC10713391; doi:10.18632/aging.205221)
Supplement: Supplementary Figure 1 [file aging-15-205221-s001.pdf]

SUPPLEMENTARY FIGURE

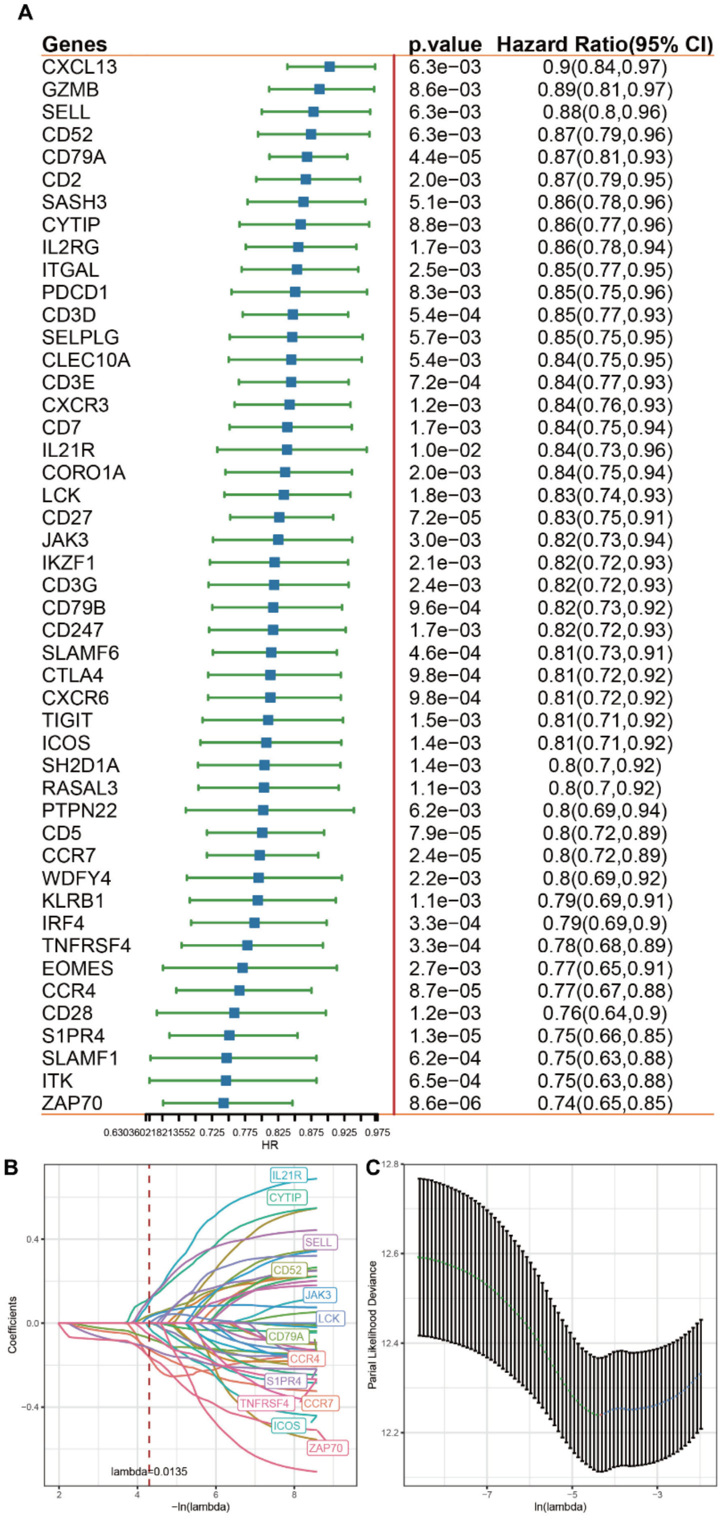

Supplementary Figure 1. Establishment of a prognostic signature. (**A**) Univariate Cox regression analysis of genes correlated with HNSC prognosis. (**B**, **C**) LASSO analysis for significant genes associated with HNSC prognosis.
